# Quinia and Amyl Nitrite in Pertussis

**Published:** 1879-12

**Authors:** 


### EXCERPTA.

---

Quinia and Amyl Nitrite in Pertussis.—Dr. E. D. Emack, of Trackville, Pa. (in *Trans. Med. Soc. Penn.*, 1879), says that during the summer of 1877 his attention was attracted to an article by Dr. George Bayles, of New York, in a Southern journal (*Va. Med. Monthly*, Aug., 1877) commending the use of amyl nitrite in conjunction with quinia in the treatment of pertussis. During the epidemic just then ending, Dr. Emack had used all the old palliatives, such as belladonna, hydrocyanic acid, nitrate of silver, chloral, the bromides in conjunction with expectorants, etc., without doing any perceptible good in curing the disease. Since seeing Dr. Bayles' article, he has had eight cases of whooping cough in which he has used the amyl and quinia; and the results "prove, beyond the shadow of a doubt, the great merit of Dr. Bayles' method." He did not, in any of the great cases, commence the amyl treatment until the characteristic inspiratory whoop was so thoroughly developed as to render a mistake in diagnosis impossible. When his directions were thoroughly complied with, the disease was invariably cured within a week; but in two of the cases, where vigilance was too early relaxed, the cough continued for nearly two weeks. Further, in none of the cases so treated, was there after the early use of the amyl, any of the usual complications, such as bronchitis, pneumonitis and cerebral convulsions, which hitherto have rendered this disease, in a large proportion of cases, fatal. He believes, with Dr. Bayles, that "in quinia there appears to be real antidotal action to the specific, root-element of the disease—whatever that may be." And he holds that the amyl nitrite "is pre-eminently a relaxer of spasm in all involuntary muscular fibre; and that, through its influence over the vaso-motor system, in allaying arterial tension and in producing peripheral deviation, it relieves any and all cerebral and spinal congestion, and thus controls the paroxysmal habit Indeed, I consider the nitrite of amyl no less a specific in the treatment of pertussis than are the alkalies in rheumatism, or quinia in malarial fever."

The doses of quinia should be proportioned to the age of the patient, every two or three hours; and to deprive it of its exceedingly bitter taste, it may be combined with a grain or two of tannic acid, using simple elixir as a vehicle. The amyl is best administered by dropping one to four drops in a teacup; and as each whooping spell approaches, let the child inhale it. The paroxysm is broken as if by magic.

